# Supplementary material for: Speg interactions that regulate the stability of excitation-contraction coupling protein complexes in triads and dyads
Source: Commun Biol. 2023 Sep 14;6:942. doi: 10.1038/s42003-023-05330-y (PMC10502019; doi:10.1038/s42003-023-05330-y)
Supplement: Supplementary file 2 — Supplementary Information [file 42003_2023_5330_MOESM2_ESM.pdf]

Supplementary Tables and Figures

**Supplementary Table 1 cardiac echo**	**n**	**LVPWs mm**	**LVPWd mm**	**LVIDs mm**	**LVIDd mm**	**LVAWs mm**	**LVAWd mm**	**EF %**	**FS %**
WT male (13-14 weeks)	6	1.30 ±0.08	0.83 ±0.06	2.13 ±0.06	3.41 ±0.04	1.48 ±0.08	0.83 ±0.06	68.2 ±3.2	37.6 ±2.3
HA-Speg male (13-14 weeks	10	1.41 ±0.10	1.04 ±0.11	2.21 ±0.07	3.28 ±0.09	1.57 ±0.09	1.0 2±0.08	64.6 ±1.7	34.6 ±1.3
WT female (13-14Weeks	5	1.25 ±0.09	0.8 4±0.07	2.03 ±0.21	3.23 ±0.08	1.23 ±0.09	0.83 ±0.06	57.9 ±6.4	37. 3±5.0
HA-Speg female (13-14weeks	4	1.16 ±0.08	0.76 ±0.06	2.02 ±0.09	3.28 ±0.09	1.25 ±0.05	0.82 ±0.06	69.1 ±4.8	38.2 ±3.7
Control (*Speg*$^{fl/fl}$) (9-10 weeks)	4	1.15 ±0.07	0.80 ±0.07	2.88 ±0.05	4.11 ±0.05	1.39 ±0.08	0.91 ±0.06	58.9 ±3.6	31.0 ±2.5
Speg-KO male (9-10 weeks)	4	0.83 ±0.02**	0.76 ±0.07	4.78 ±0.94*	5.30 ±0.82*	0.8 1±0.09**	0.66 ±0.09	24.4 ±6.5**	11.4 ±3.1**
Control (9-10 weeks)	6	1.31 ±0.04	0.99 ±0.05	2.64 ±0.11	3.78 ±0.15	1.37 ±0.04	0.91 ±0.04	62.4 ±1.2	33.2 ±0.8
Speg-KO female (9-10 weeks)	5	0.79 ±0.09***	0.66 ±0.07**	3.59 ±0.06***	4.2 0±0.10	0.84 ±0.01***	0.64 ±0.02**	29.7 ±3.5***	13.9 ±1.8***

Supplementary Table 1. Cardiac Echo values

**Supplementary Table 2 Body composition**	**n**	**Weight g**	**Length mm**	**Bone area cm^2**	**Fat weight g**	**Lean weight g**	**% Lean**	**%fat**	**BMC g**	**BMD mg/cm^2**
WT male (10 weeks	8	24.6 ±0.7	97.6 ±0.6	7.99 ±0.14	8.0 0±0.57	16.0 ±0.4	65.4 ±1.6	32.4 ±1.6	0.55 ±0.01	69.1 ±1.2
HA-Speg male (10 weeks)	8	22.7 ±0.4*	96.0 ±0.4*	7.74 ±0.09	7. 6±0.28	14.7 ±0.30*	64.5 ±0.92	33.2 ±0.9	0.5 2±0.02	67.1 ±1.5
WT female (10 Weeks)	7	17.8 ±0.6	93.5 ±0.7	7.96 ±0.20	6.30 ±0.33	11.5 ±0.5	62.8 ±1.4	34.4 ±1.4	0.53 ±0.02	66.1 ±0.7
HA-Speg female (10 weeks)	7	18.1 ±0.2	91.9 ±0.1	7.56 ±0.06	6.03 ±0.20	11.6 ±0.2	64.1 ±0.9	33.3 ±0.9	0.47 ±0.01	61.5 ±0.6
Control male (*Speg$^{fl/fl}$*) (9-10 weeks)	7	23.8 ±0.6	91.9 ±0.5	7.75 ±0.09	7.22 ±0.35	16.1 ±0.6	62.7 ±1.1	31.1 ±1.5	0.51 ±0.01	66.3 ±1.6
Speg-KO male (9-10 weeks)	4	17.3 ±0.4***	87.8 ±0.7**	6.95 ±0.14**	5.44 ±0.22*	11.5 ±0.14***	61.4 ±0.3	32.1 ±0.6	0.37 ±0.01***	52.8 ±0.9***
Control female (*Speg$^{fl/fl}$*) (9-10 weeks)	4	19.1 ±0.7	87.6 ±0.2	7.38 ±0.38	5.11 ±0.48	11.3 ±0.3	59.4 ±0.2	26.7 ±0.2	0.42 ±0.03	57.3 ±1.8
Speg-KO female (9 weeks)	4	12.5 ±0.9**	81.5 ±2.5	6.67 ±0.07	3.52 ±0.39*	7.00 ±0.43***	56.1 ±0.4	27. 9±1.4	0.34 ±0.02	50.2 ±2.5

Supplementary Table 2. Body Composition

Antibody list

Antibody	Supplier	Catalog #	Animal source	Application	Publication (PMID) / Validation
$Ca_V1.2\alpha1$	Alomone labs	ACC-003	Rabbit	Western blot (1:1,000)	21408028; 37415128; 37128962
$Ca_V1.1\alpha1$	Thermo Scientific	MA3-920	Mouse	Western blot (1:1,000)	29368397; 28337975; 27669143
HA-conjugated beads	Thermo Scientific	88836	Magnetic beads	IP (10 μl/100 μg lysates)	28666126; 21508667
Jph1 (aa 559-572)	ThermoFisher	40-5100	Rabbit	Western blot (1:1,000)	29368397; 23148318; 26405035
Jph2 (aa 1-50)	Invitrogen	MA5-32864	Mouse	Western blot (1:1,000)	Validated by WB in the mouse heart (By Invitrogen)
Jph2 (aa 565-580)	Invitrogen	40-5300	Rabbit	WB (1:1,000)/IP (1 μg/100 μg lysates)	34524407; 32697997; 30786093
Jph2 (aa 431-680)	Santa Cruz	sc-377086	Mouse	Western blot (1:500)	34401916; 31217359; 28740174
RyRs	DSHB	34C-c	Mouse	WB (1:500)/IP (1 μg/100 μg lysates)	33956073; 30147660; 32681036
Speg	Sino Biological	12472-T16	Rabbit	Western blot (1:1,000)	33355670; 35763354; 37162921
MyHC I	DSHB	BA-F8	Mouse	Fiber typing (1:200)	33198807; 30866899; 21697544
MyHC IIa	DSHB	SC-71	Mouse	Fiber typing (1:200)	33198807; 30081940; 28982947
MyHC IIb	DSHB	BF-F3	Mouse	Fiber typing (1:200)	33198807; 30081940; 28982947
Laminin	abcam	Ab11575	Rabbit	Fiber typing (membrane, 1:500)	33198807; 32358797; 35665306
Fsd2	Proteintech	25609-1-AP	Rabbit	WB (1:1000)/IP (1 μg/100 μg lysates)	Validated by WB in the mouse heart (By Proteintech)
Esd	Invitrogen	PA5-96537	Rabbit	Western blot (1:1000)	Validated by WB in the mouse kidney and cell lines (By Proteintech)
αHA	Proteintech	66006-2-Ig	Mouse	Western blot (1:1000)	36179025; 33811247; 32951003

Supplementary Table 3. Antibodies used

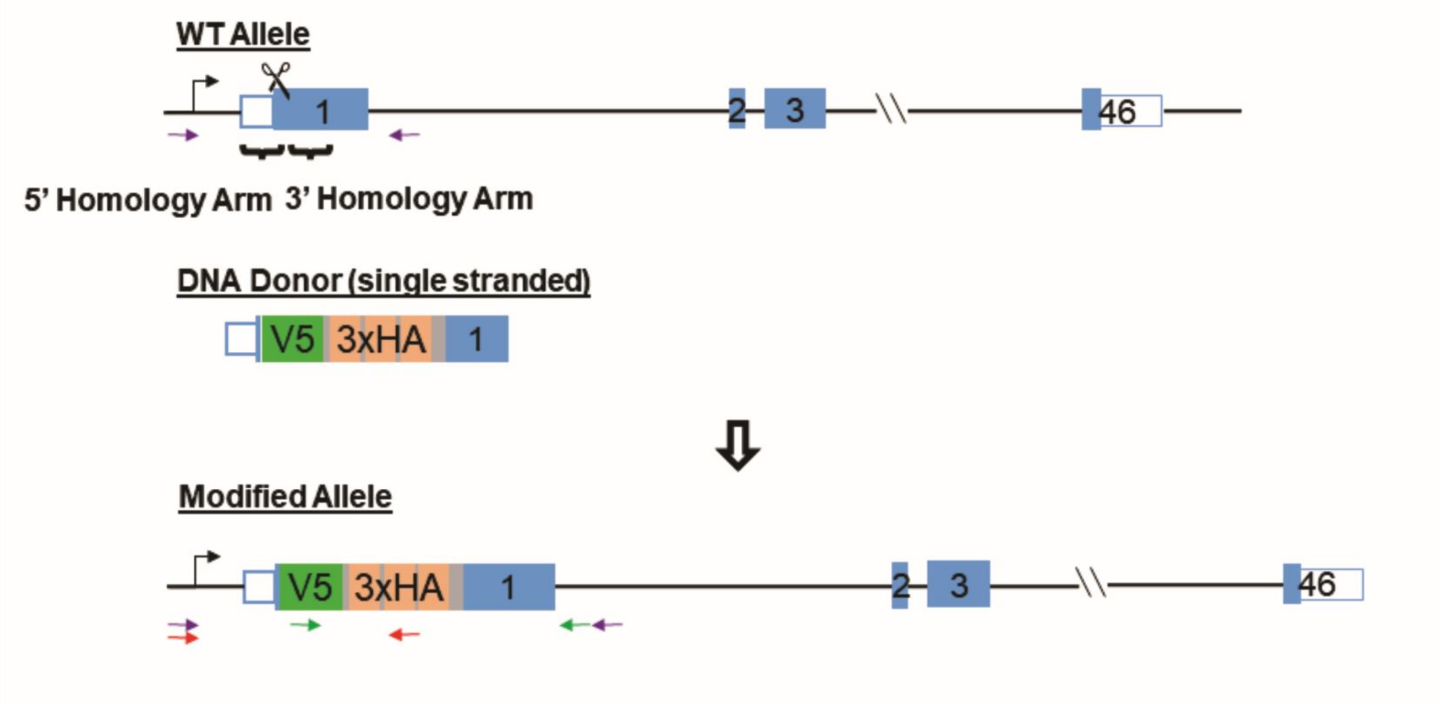

Supplementary Fig. 1 Allele cartoon depicting CRISPR/Cas9-initiated design to target a V5-3xHA tag to the N-terminus of *Speg.* A guide RNA target was selected based on its proximity of the cut site to the ATG methionine codon to initiate translation. Homology arms approximately 45 nucleotides in length were selected flanking the break between the first and second amino acids. The V5 (green box) and HA (orange box) sequences were flanked by flexible G-S linker sequences (grey boxes). The modified allele was verified using standard PCR, with primers flanking the inserted sequence (purple) and internal to the targeted allele (5' red, 3' green).

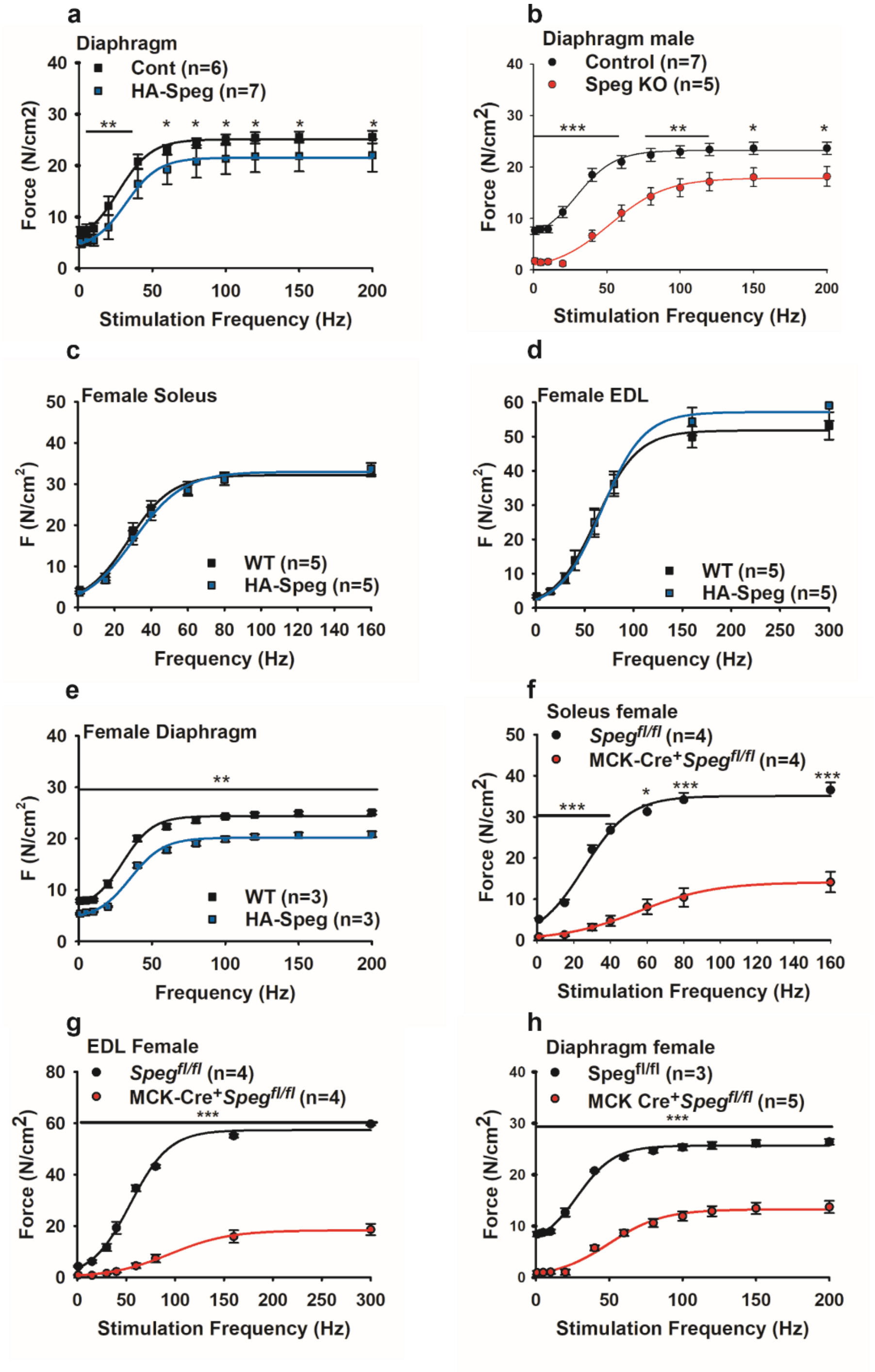

Supplementary Figure 2. Force generation in HA-Speg, Speg KO and Control female mice. Skeletal muscle function in HA-Speg (14 weeks), WT (13 weeks), Speg-KO (8-9 weeks) and *Speg*$^{fl/fl}$ (8-9 weeks) female mice were assessed using skeletal muscle force frequency measurements. The Speg-KO mice used were younger than the HA-Speg mice because they become extremely sick at around 10 weeks of age and often do not survive the echoes. Echoes on HA-Speg mice and age matched WT controls (13 weeks of age) were performed as described in Methods. **A.** Force Frequency data for WT and HA-Speg female soleus. **B.** Force Frequency data for female EDL from control and HA-Speg. **C.** Force Frequency data for WT and HA-Speg female diaphragm. **D.** Force Frequency data for Control and Speg KO female soleus. **E.** Force Frequency data for WT and Speg-KO female EDL. **F.** Force Frequency data for Control and Speg-KO female diaphragm. Data are plotted as mean ± SEM. ***P<0.001, **P<0.01, and *P<0.05.

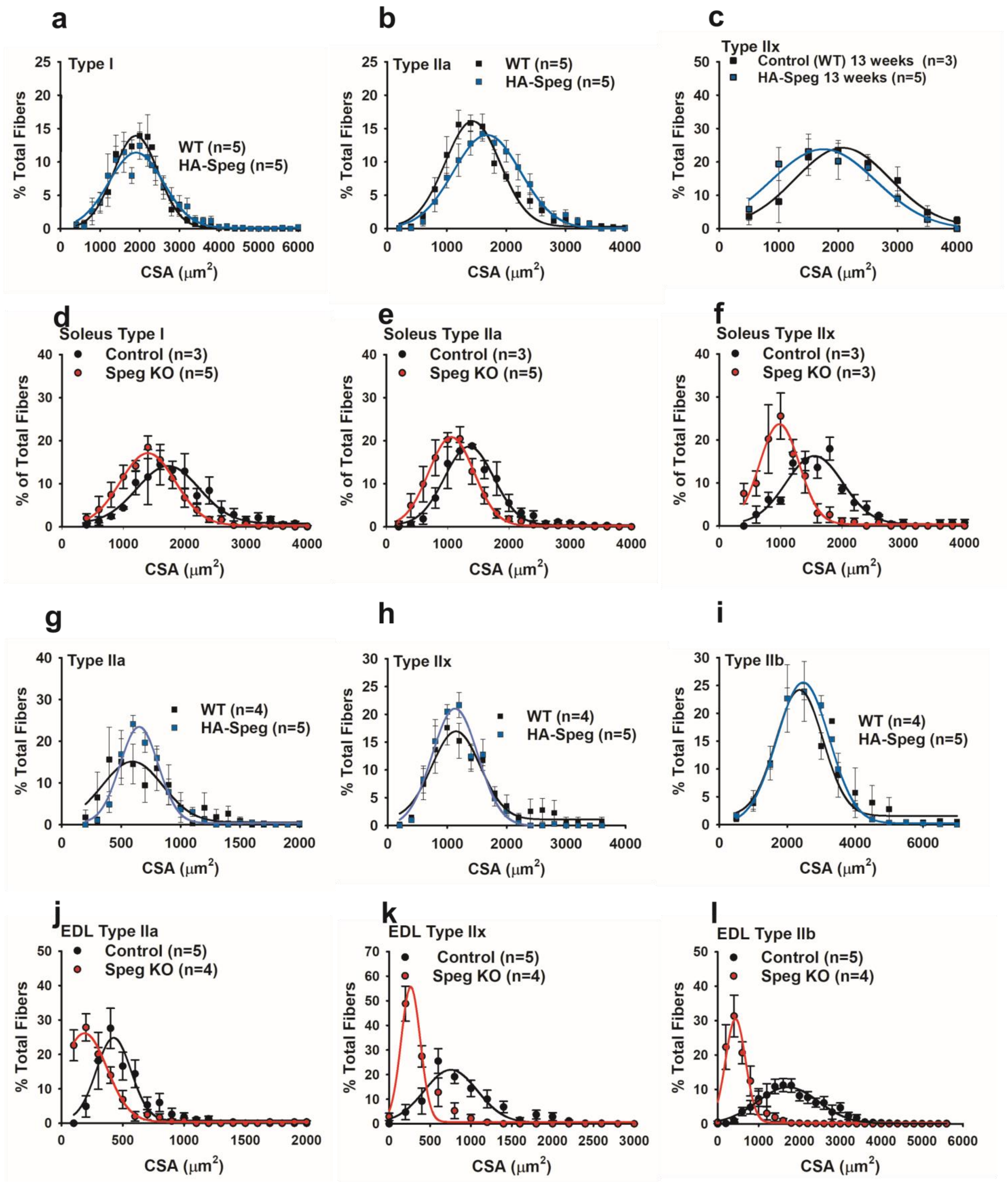

Supplementary Figure 3. Effect of Speg deficiencies on the distribution of CSA of different fiber types. Soleus and EDL muscle from WT (control), HA-Speg, Spegfl/fl (control) and Speg KO mice were prepared, labeled with fiber type specific antibodies and analyzed with MyoSight. **A-C** Binned CSA distributions of Type I, IIa and IIx fibers, respectively in soleus of HA-Speg. **D-F** Binned CSA distributions of Type I, IIa and IIx fibers, respectively in soleus of Speg KO. **G-H.** Binned CSA distributions of Type IIa, IIx and IIb fibers, respectively in

EDL of HA-Speg mice. **J-K.** Binned CSA distributions of Type IIa, IIx and IIb fibers, respectively in EDL of **Speg KO mice.**

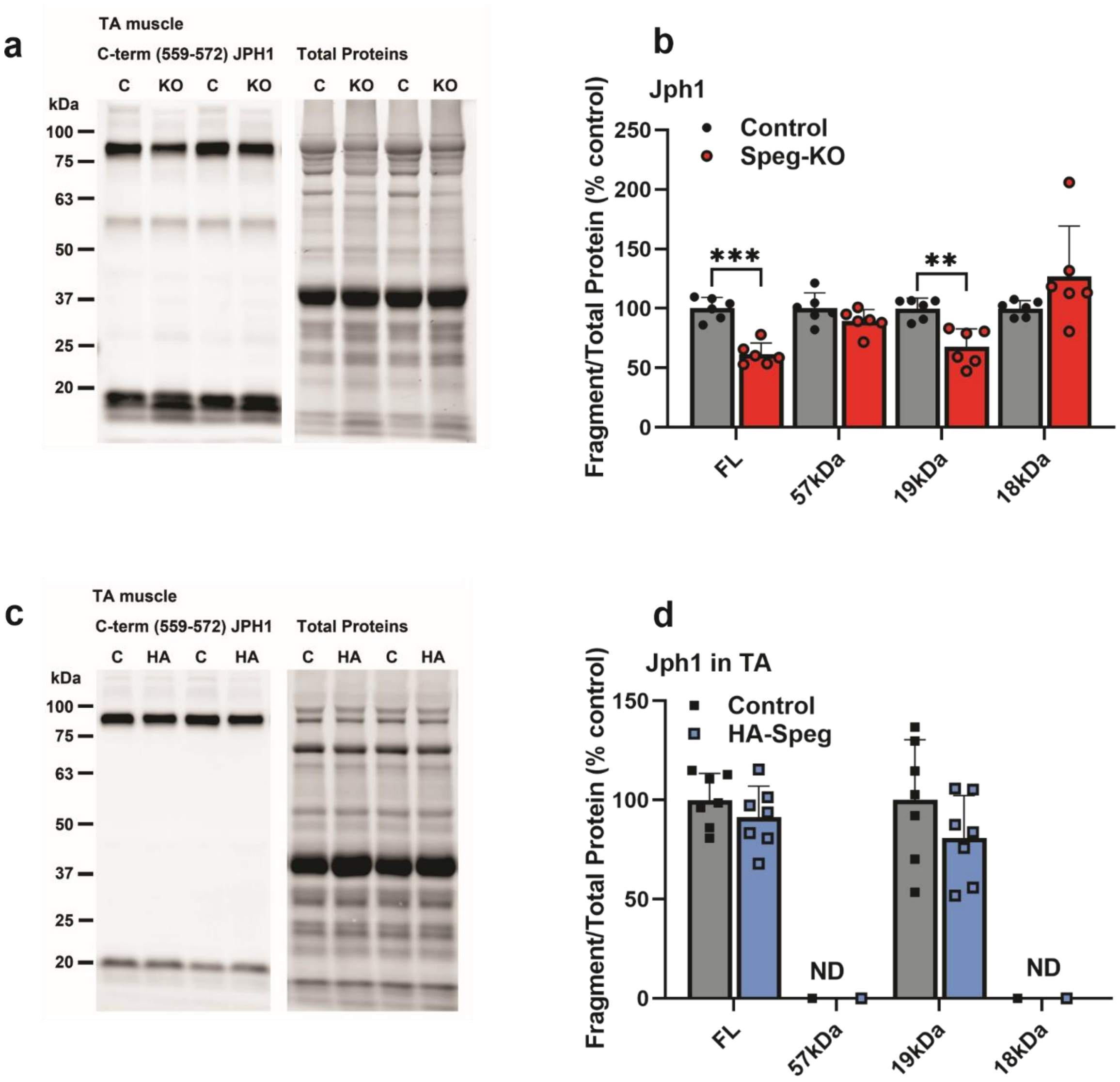

Supplementary Figure 4. Jph1 fragmentation in Speg Deficient Mice. A. Western blot in TA homogenates from control and Speg KO mice with an antibody to Jph1. Left panel western blot. Right panel protein. **B.** Quantitation of Fragments of Jph1 (C=6, KO n=6). **C** Western blot in TA homogenates from control and HA-Speg mice with an antibody to Jph1 (C=7, HA=7). Left panel western blot. Right panel protein. **D.** Quantitation of fragments of Jph1. Mean ± SD. ***P<0.001, **P<0.01, *P<.0.05

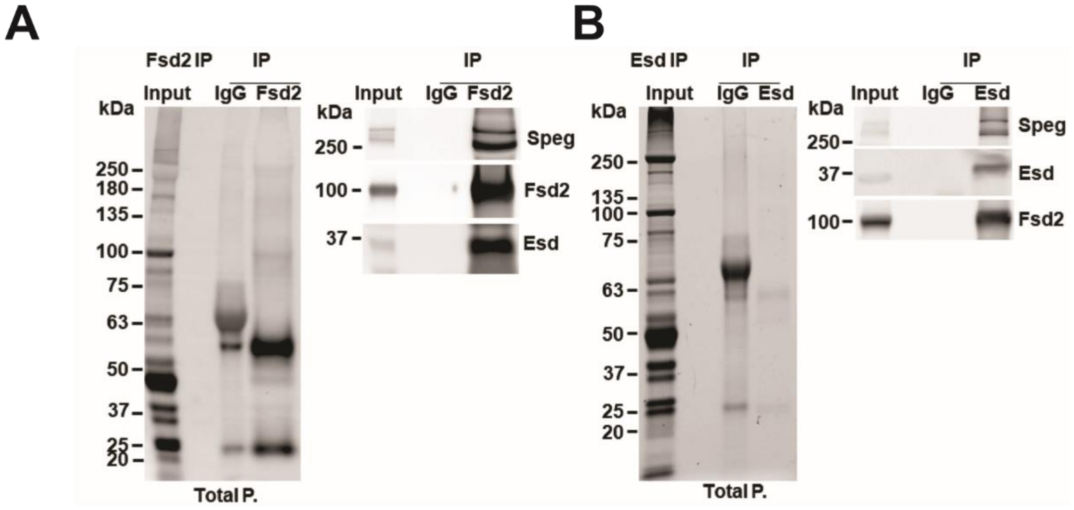

Supplementary Figure 5. IPs of Fsd2 and Esd. A. Immunoprecipitation of Fsd2 from gastrocnemius muscle of WT mice followed by western blotting with antibodies to Speg, Fsd2 and Esd. **A**. Immunoprecipitation of Esd from gastrocnemius muscle of WT mice followed by western blotting with antibodies to Speg, Fsd2 and Esd.

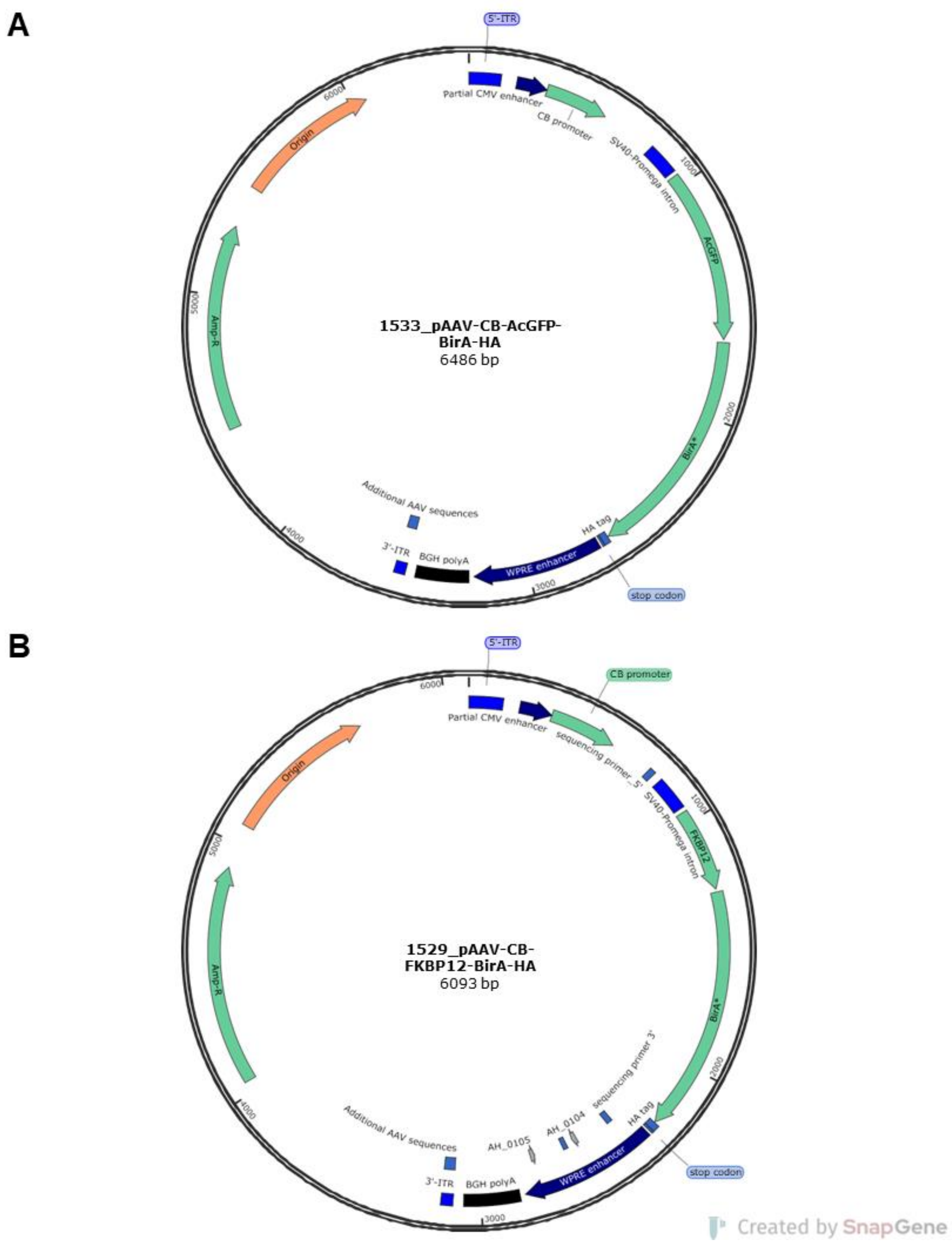

Supplementary Figure 6. A. Plasmid “1533_pAAV-CB-AcGFP-BirA-HA” which expresses an *Aequorea coerulescens* GFP (AcGFP) with a Gly-Gly-Ser-Gly flexible linker fused to a C-terminal BirA* biotin ligase followed by an Ala and a Hemagglutinin (HA) epitope tag. The transgene cassette is driven by a Chicken beta Actin promoter with a CMV enhancer element and includes a 3’ woodchuck hepatitis virus posttranscriptional regulatory element (WPRE) and bovine growth hormone polyadenylation signal downstream. B. A plasmid in the identical configuration named “1529_pAAV-CB-FKBP12-BirA-HA.”
