# Supplementary material for: Speg interactions that regulate the stability of excitation-contraction coupling protein complexes in triads and dyads
Source: Commun Biol. 2023 Sep 14;6:942. doi: 10.1038/s42003-023-05330-y (PMC10502019; doi:10.1038/s42003-023-05330-y)
Supplement: Supplementary file 3 — Description of Additional Supplementary Files [file 42003_2023_5330_MOESM3_ESM.pdf]

Description of Additional Supplementary Files

File name: Supplementary Data 1
Description: Proteomic datasets 1-12.

File name: Supplementary Data 2
Description: Source data for all manuscript figures.

File name: Supplementary Data 3
Description: Source data for Supplementary Tables and Figures.

File name: Supplementary Data 4
Description: Uncropped gels.
