# Supplementary material for: Speg interactions that regulate the stability of excitation-contraction coupling protein complexes in triads and dyads
Source: Commun Biol. 2023 Sep 14;6:942. doi: 10.1038/s42003-023-05330-y (PMC10502019; doi:10.1038/s42003-023-05330-y)
Supplement: Supplementary file 7 — Supplementary Data 4 [file 42003_2023_5330_MOESM7_ESM.pdf]

Figure 1a

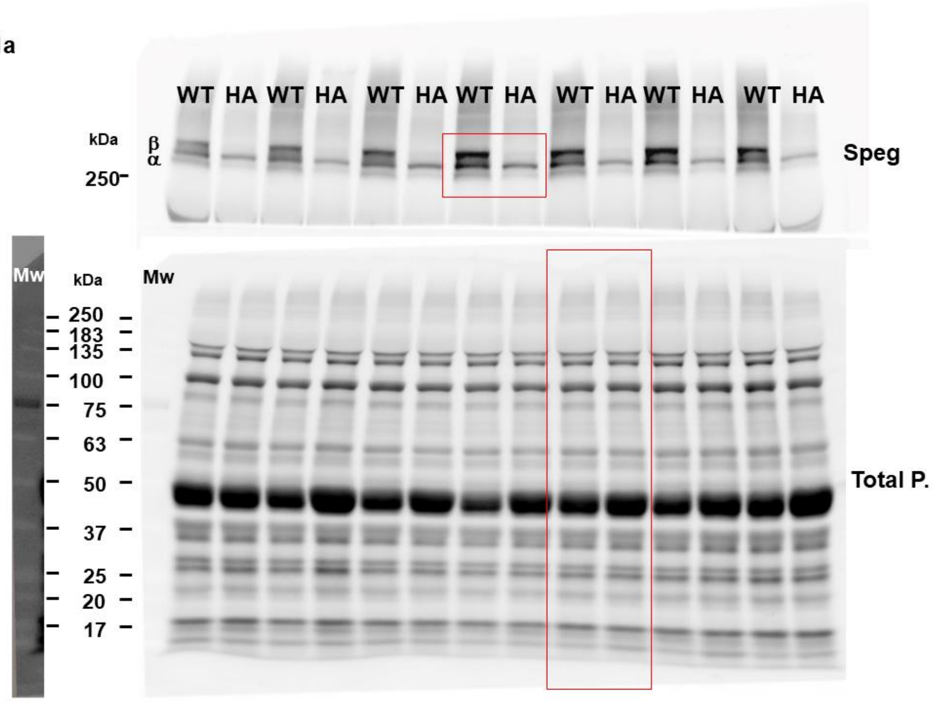
WT HA WT HA WT HA WT HA WT HA WT HA WT HA
kDa
β
α
250
Speg
Mw
kDa
Mw
250
183
135
100
75
63
50
37
25
20
17
Total P.

Figure 1b

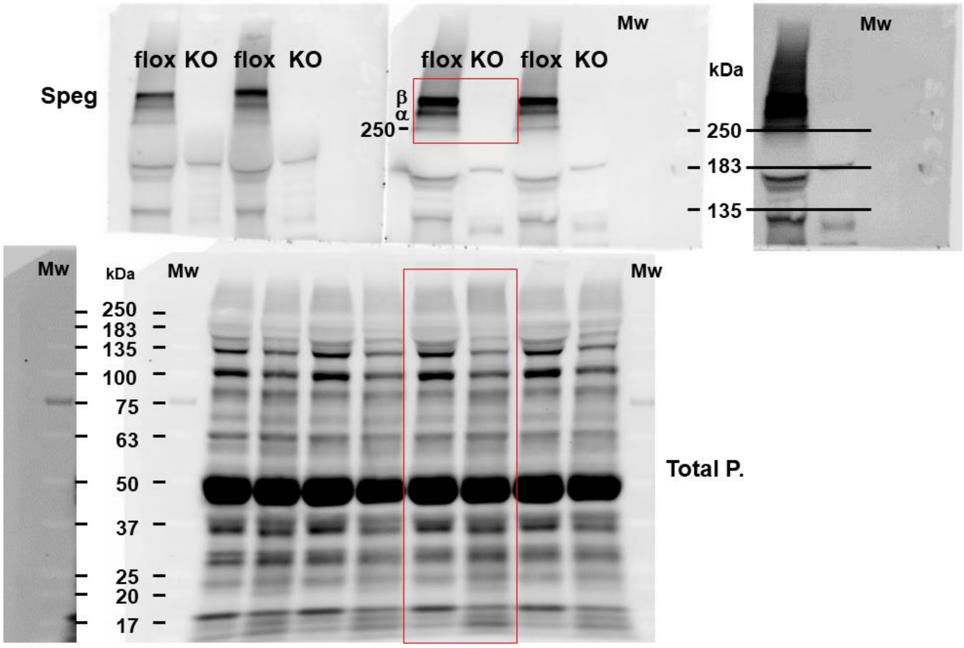

Speg
flox KO flox KO
flox KO flox KO
Mw
β
α
250
kDa
Mw
250
183
135
Mw
kDa
Mw
250
183
135
100
75
63
50
37
25
20
17
Mw
Total P.

Figure 1c

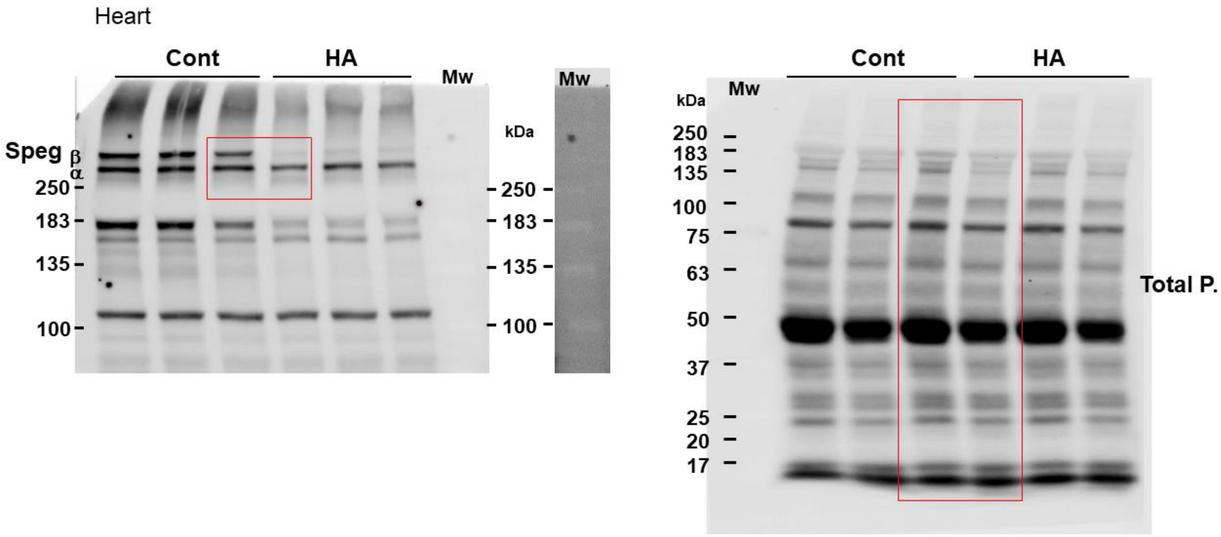
Heart
Cont
HA
Mw
Mw
kDa
Speg
β
α
250
183
135
100
250
183
135
100
Cont
HA
Mw
kDa
250
183
135
100
75
63
50
37
25
20
17
Total P.

Figure 1d

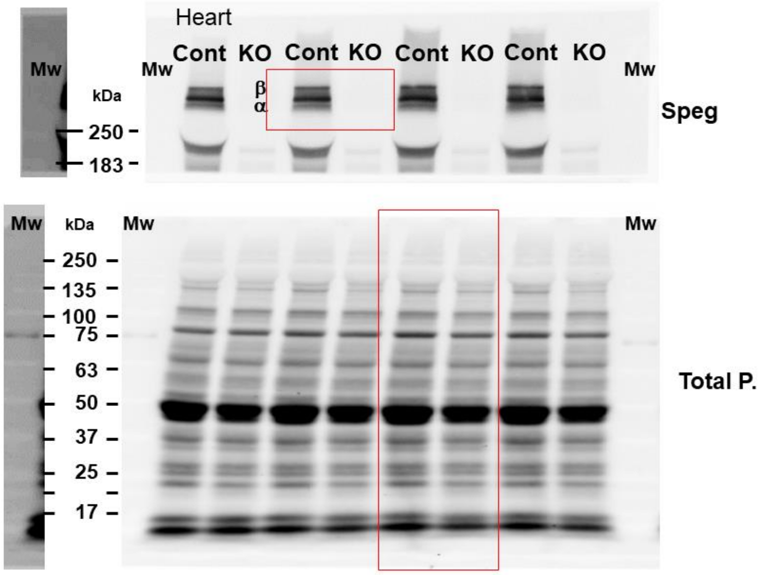

Heart
Cont KO Cont KO Cont KO Cont KO
Mw
kDa
β
α
250
183
Speg
Mw
kDa
250
135
100
75
63
50
37
25
17
Total P.

Figure 1g

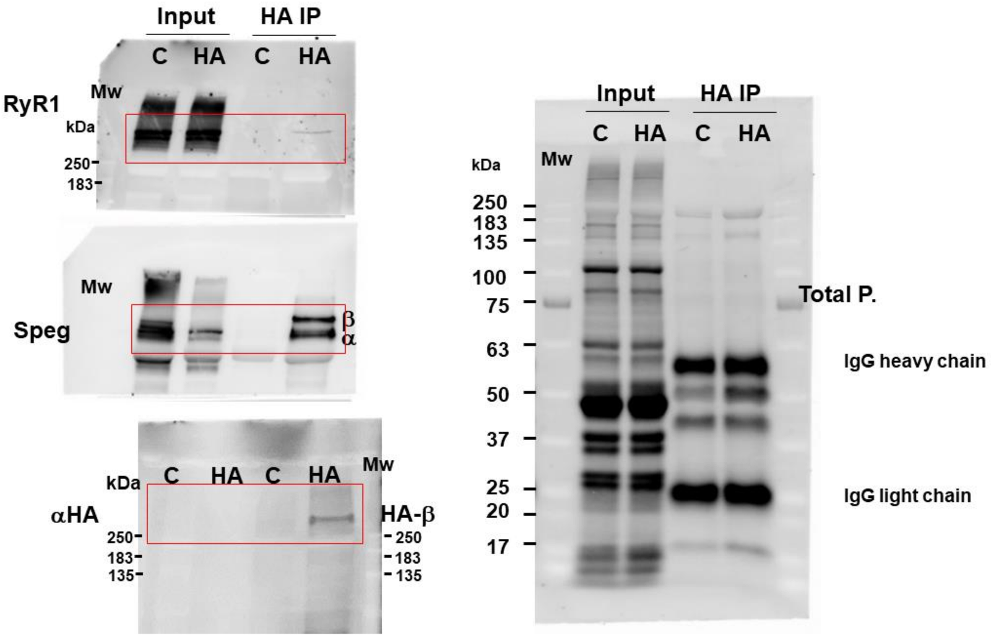
Input
HA IP
C
HA
C
HA
RyR1
Mw
kDa
250
183
Mw
Speg
β
α
kDa
C
HA
C
HA
Mw
αHA
HA-β
250
183
135
250
183
135
Input
HA IP
C
HA
C
HA
kDa
Mw
250
183
135
100
75
63
50
37
25
20
17
Total P.
IgG heavy chain
IgG light chain

Figure 1h

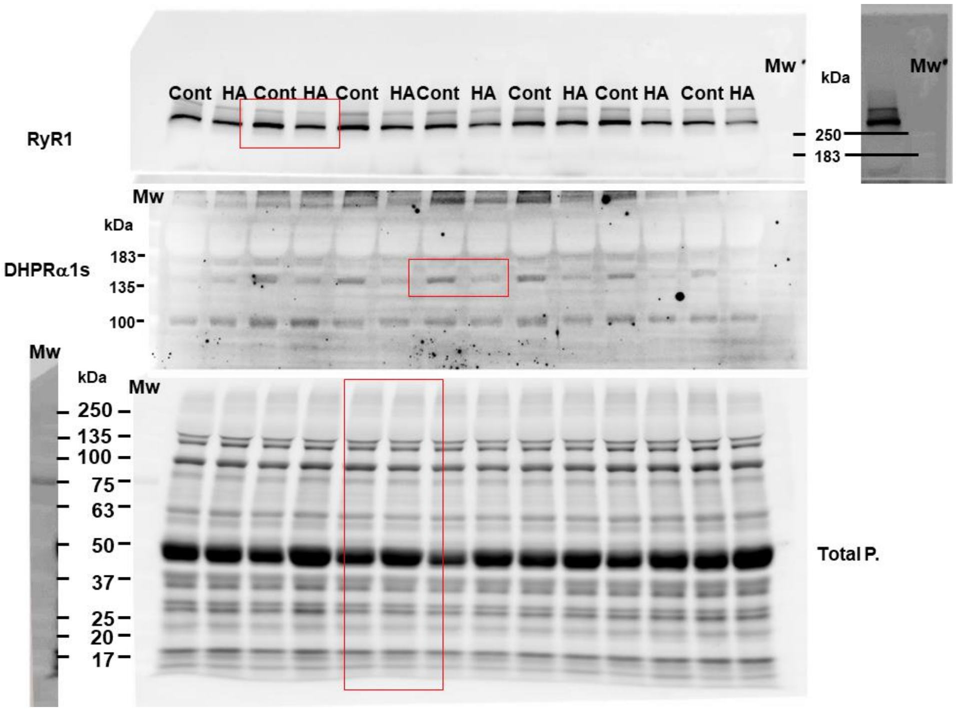

Cont HA Cont HA Cont HA Cont HA Cont HA Cont HA Cont HA
Mw
kDa
250
183
RyR1
DHPRα1s
183
135
100
Total P.
250
135
100
75
63
50
37
25
20
17

Figure 1i

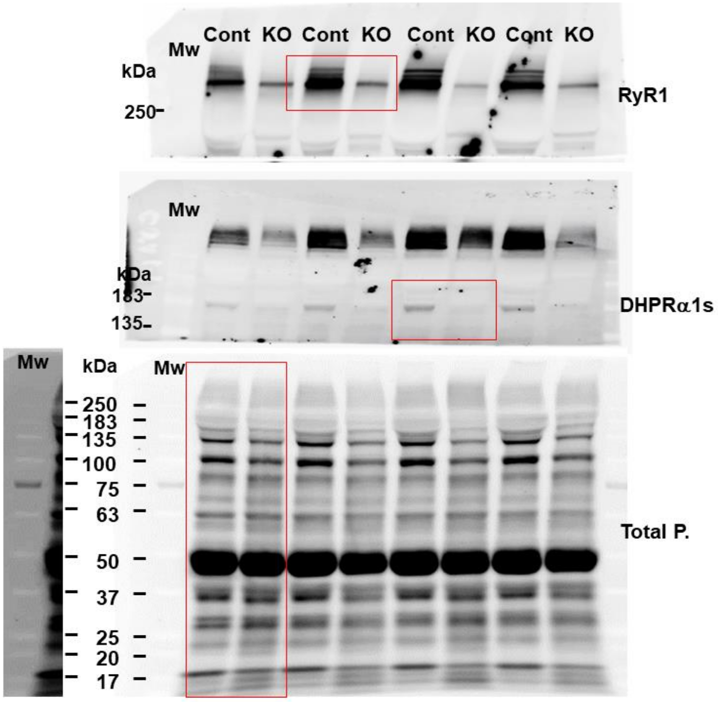
Mw
Cont
KO
Cont
KO
Cont
KO
Cont
KO
kDa
250
RyR1
Mw
kDa
183
135
DHPRα1s
Mw
kDa
Mw
250
183
135
100
75
63
50
37
25
20
17
Total P.

Figure 1j

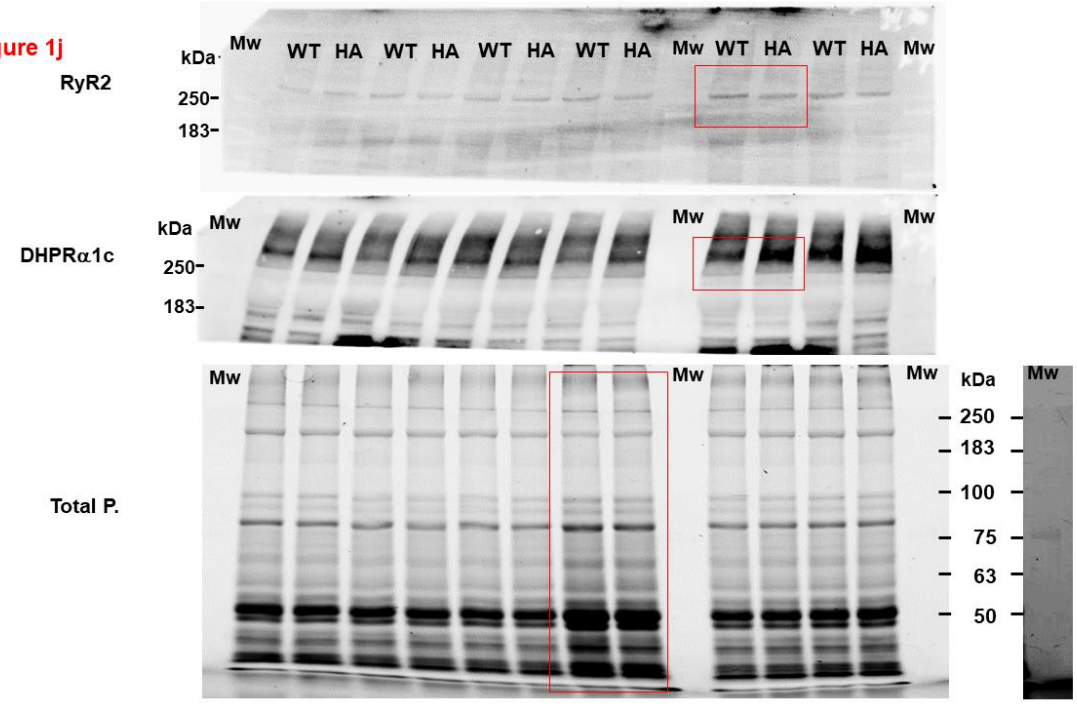

RyR2
kDa
Mw WT HA WT HA WT HA WT HA Mw WT HA WT HA Mw
250
183
DHPRα1c
kDa
Mw
Mw
Mw
250
183
Total P.
Mw
Mw
Mw
kDa
250
183
100
75
63
50
Mw

Figure 1k

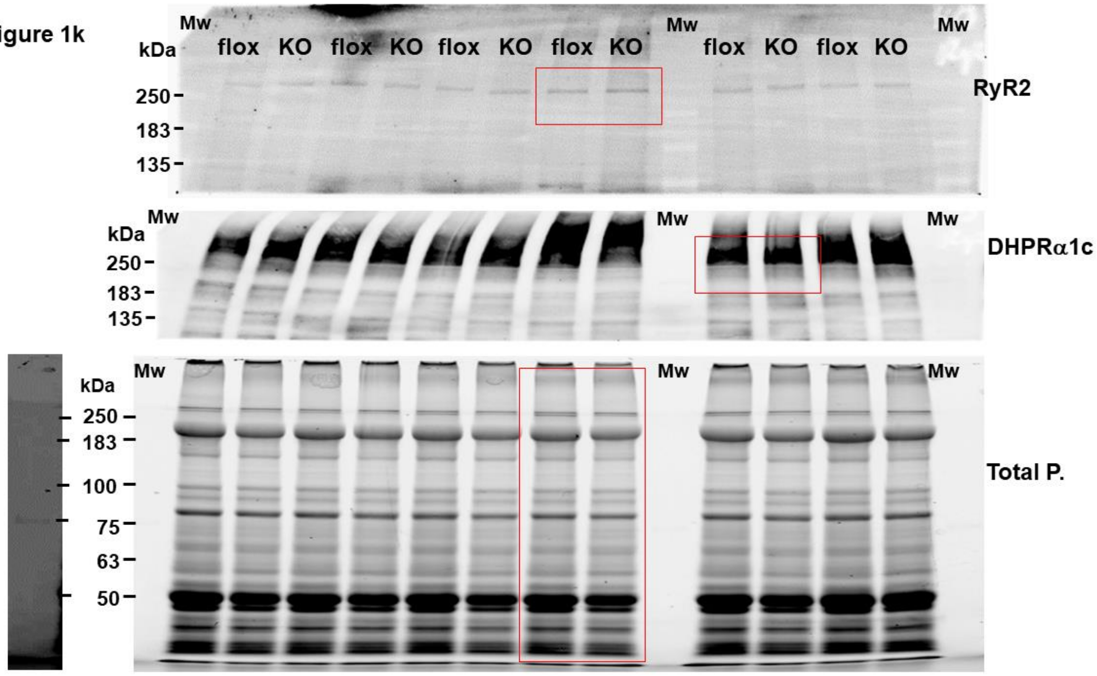
Mw
kDa
flox KO flox KO flox KO flox KO
Mw
flox KO flox KO
Mw
250
183
135
RyR2
Mw
kDa
250
183
135
Mw
Mw
DHPRα1c
Mw
kDa
250
183
100
75
63
50
Mw
Mw
Total P.

Figure 4a

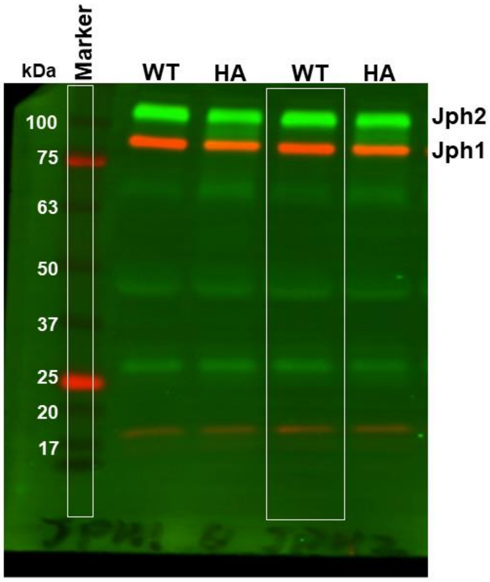

Figure 4b

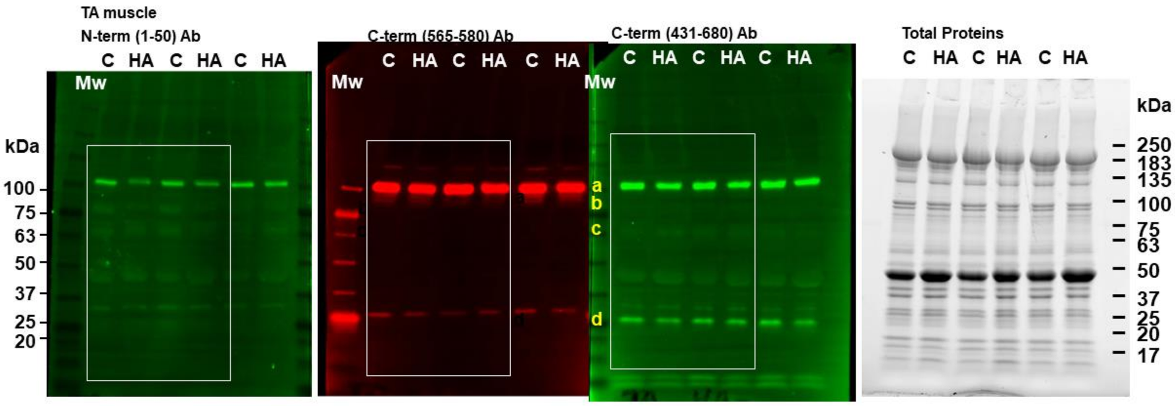
TA muscle
N-term (1-50) Ab
C HA C HA C HA
Mw
kDa
100
75
63
50
37
25
20
C-term (565-580) Ab
C HA C HA C HA
Mw
C-term (431-680) Ab
C HA C HA C HA
Mw
a
b
c
d
Total Proteins
C HA C HA C HA
kDa
250
183
135
100
75
63
50
37
25
20
17

Figure 4d

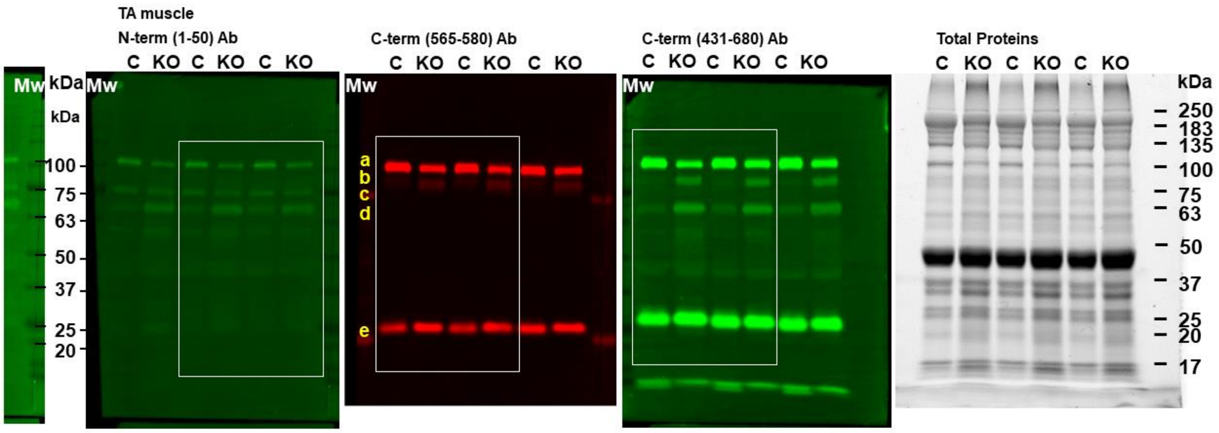
TA muscle
N-term (1-50) Ab
C KO C KO C KO
C-term (565-580) Ab
C KO C KO C KO
C-term (431-680) Ab
C KO C KO C KO
Total Proteins
C KO C KO C KO
Mw
kDa
100
75
63
50
37
25
20
a
b
c
d
e
250
183
135
17

Figure 4f

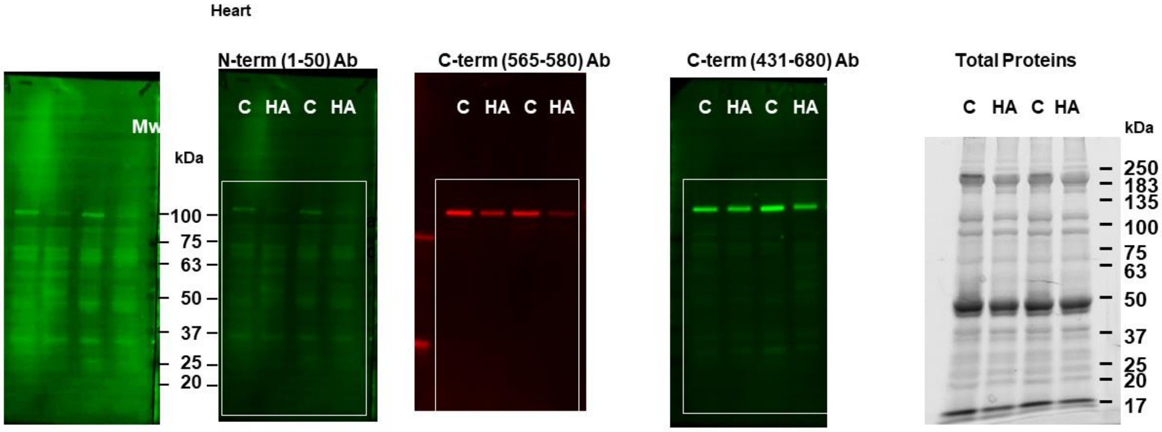
Heart
N-term (1-50) Ab
C-term (565-580) Ab
C-term (431-680) Ab
Total Proteins
Mw
kDa
C HA C HA
C HA C HA
C HA C HA
C HA C HA
100
75
63
50
37
25
20
kDa
250
183
135
100
75
63
50
37
25
20
17

Figure 4h

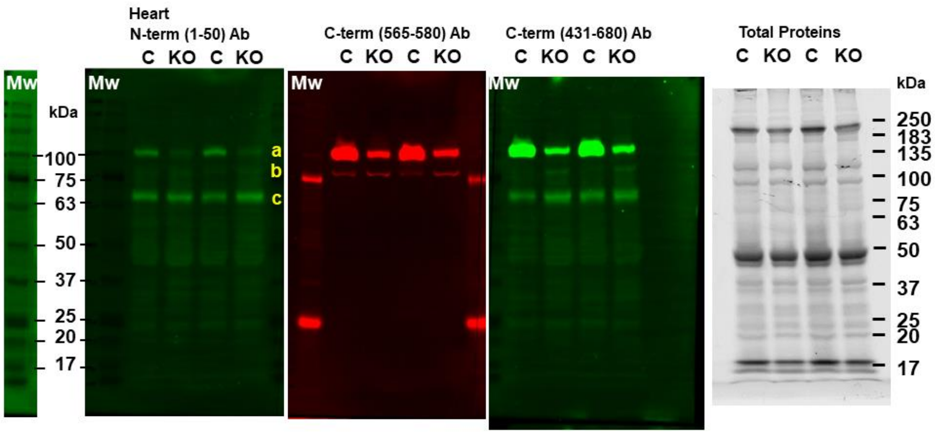
Heart
N-term (1-50) Ab
C KO C KO
C-term (565-580) Ab
C KO C KO
C-term (431-680) Ab
C KO C KO
Total Proteins
C KO C KO
Mw
Mw
Mw
Mw
kDa
100
75
63
50
37
25
20
17
a
b
c
kDa
250
183
135
100
75
63
50
37
25
20
17

Supplemental Figure5a

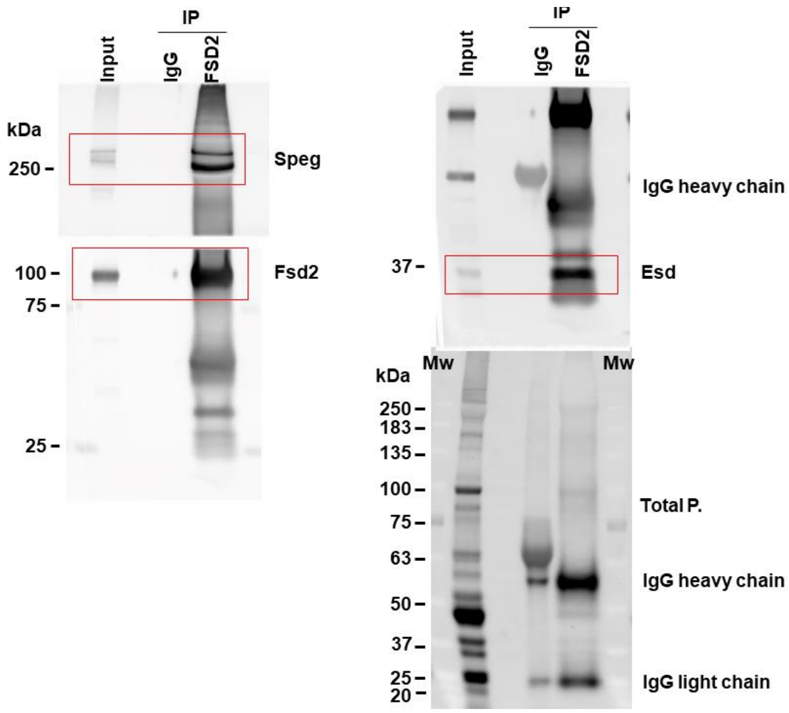

Supplemental Figure5b

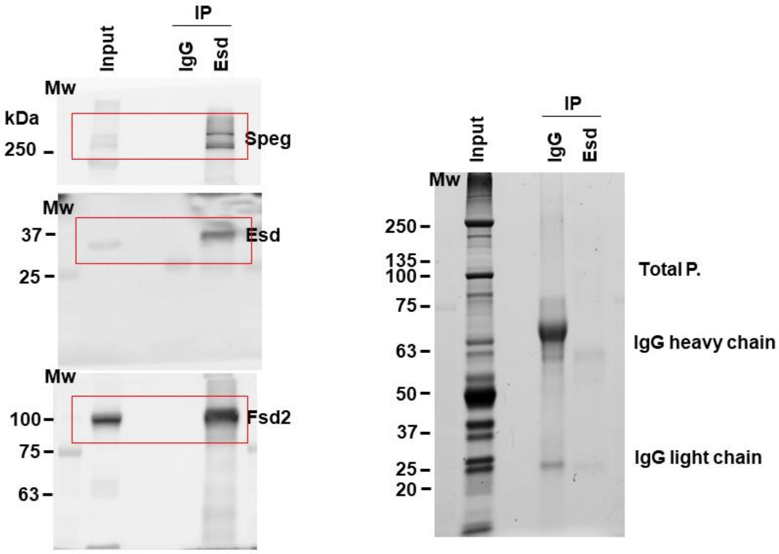

Figure 5d

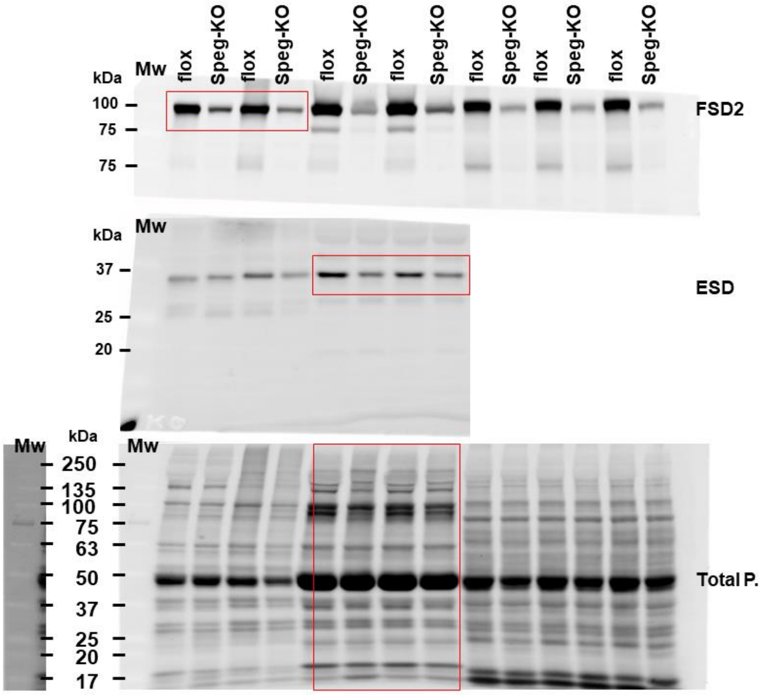

Mw
flox
Speg-KO
flox
Speg-KO
flox
Speg-KO
flox
Speg-KO
flox
Speg-KO
flox
Speg-KO
flox
Speg-KO
kDa
100
75
75
FSD2
Mw
kDa
37
25
20
ESD
Mw
kDa
Mw
250
135
100
75
63
50
37
25
20
17
Total P.

Figure 5e

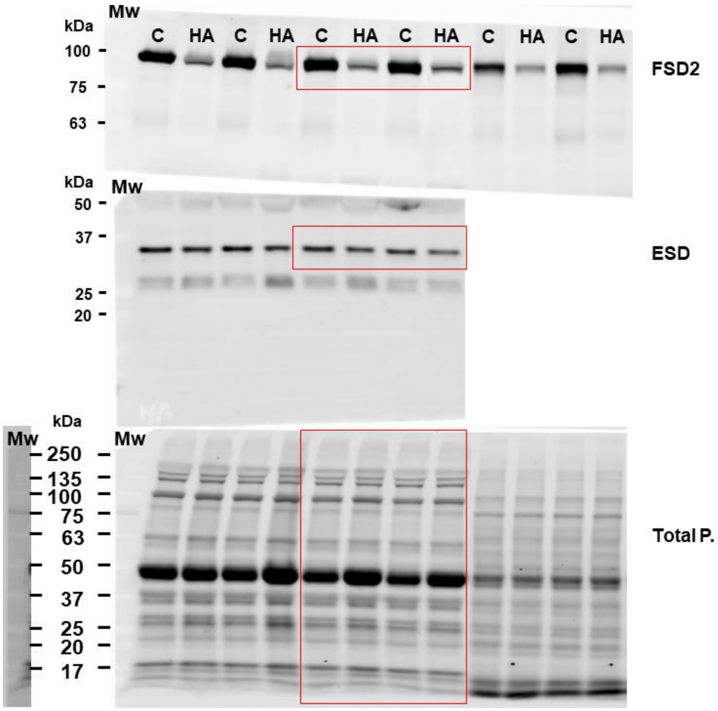

Mw
kDa
C HA C HA C HA C HA C HA C HA
100
75
63
FSD2
kDa
Mw
50
37
25
20
ESD
kDa
Mw
Mw
250
135
100
75
63
50
37
25
20
17
Total P.

Figure 6b

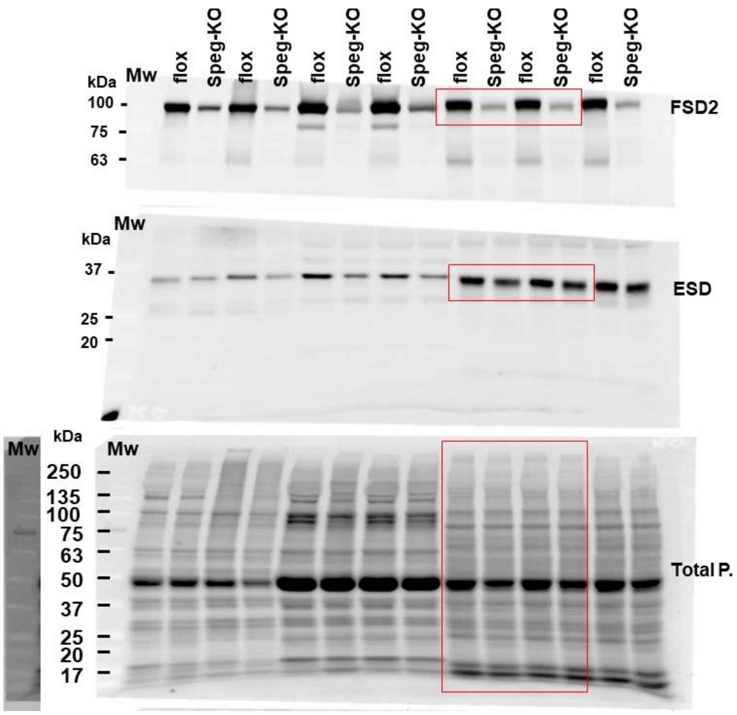
kDa
Mw
flox
Speg-KO
flox
Speg-KO
flox
Speg-KO
flox
Speg-KO
flox
Speg-KO
flox
Speg-KO
flox
Speg-KO
100
75
63
FSD2
Mw
kDa
37
25
20
ESD
Mw
kDa
Mw
250
135
100
75
63
50
37
25
20
17
Total P.

Figure 6c

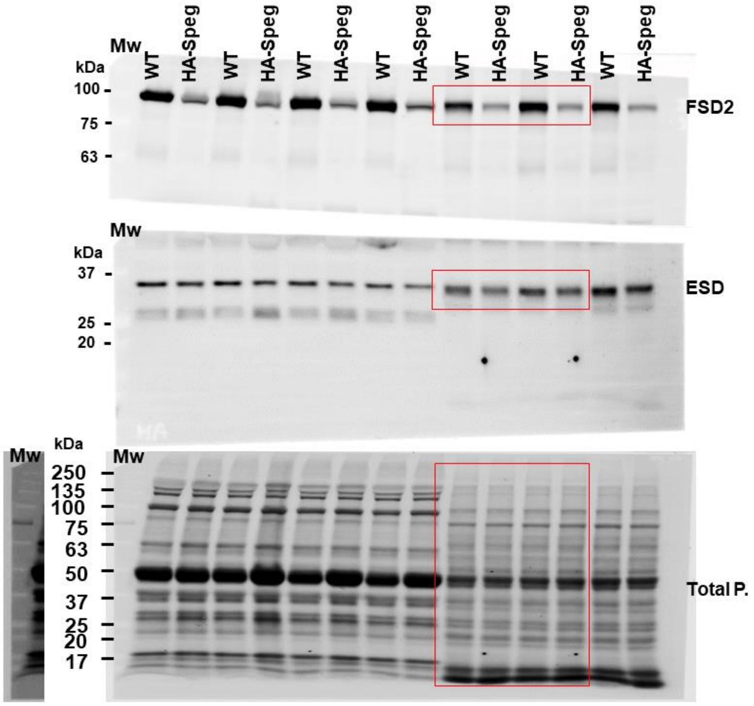
Mw
kDa
WT
HA-Speg
WT
HA-Speg
WT
HA-Speg
WT
HA-Speg
WT
HA-Speg
WT
HA-Speg
WT
HA-Speg
100
75
63
FSD2
Mw
kDa
37
25
20
ESD
Mw
kDa
Mw
250
135
100
75
63
50
37
25
20
17
Total P.
