# Supplementary material for: Speg interactions that regulate the stability of excitation-contraction coupling protein complexes in triads and dyads
Source: Commun Biol. 2023 Sep 14;6:942. doi: 10.1038/s42003-023-05330-y (PMC10502019; doi:10.1038/s42003-023-05330-y)
Supplement: Supplementary file 8 — Reporting Summary [file 42003_2023_5330_MOESM8_ESM.pdf]

nature portfolio

Corresponding author(s): SUSAN L. HAMILTON

Last updated by author(s): Sep 5, 2023

Reporting Summary

Nature Portfolio wishes to improve the reproducibility of the work that we publish. This form provides structure for consistency and transparency in reporting. For further information on Nature Portfolio policies, see our Editorial Policies and the Editorial Policy Checklist.

Please do not complete any field with "not applicable" or n/a. Refer to the help text for what text to use if an item is not relevant to your study.
For final submission: please carefully check your responses for accuracy; you will not be able to make changes later.

Statistics

For all statistical analyses, confirm that the following items are present in the figure legend, table legend, main text, or Methods section.

n/a	Confirmed	
☐	☒	The exact sample size (n) for each experimental group/condition, given as a discrete number and unit of measurement
☐	☒	A statement on whether measurements were taken from distinct samples or whether the same sample was measured repeatedly
☐	☒	The statistical test(s) used AND whether they are one- or two-sided<br>*Only common tests should be described solely by name; describe more complex techniques in the Methods section.*
☐	☒	A description of all covariates tested
☐	☒	A description of any assumptions or corrections, such as tests of normality and adjustment for multiple comparisons
☐	☒	A full description of the statistical parameters including central tendency (e.g. means) or other basic estimates (e.g. regression coefficient) AND variation (e.g. standard deviation) or associated estimates of uncertainty (e.g. confidence intervals)
☐	☒	For null hypothesis testing, the test statistic (e.g. F, t, r) with confidence intervals, effect sizes, degrees of freedom and P value noted<br>*Give P values as exact values whenever suitable.*
☒	☐	For Bayesian analysis, information on the choice of priors and Markov chain Monte Carlo settings
☒	☐	For hierarchical and complex designs, identification of the appropriate level for tests and full reporting of outcomes
☒	☐	Estimates of effect sizes (e.g. Cohen's d, Pearson's r), indicating how they were calculated

Our web collection on statistics for biologists contains articles on many of the points above.

Software and code

Policy information about availability of computer code

Data collection	Body composition (DEXA, Lunar PIXImus), Immunoblot (ChemiDoc, BioRad), RT-PCR (ViiA7, Applied Biosystems), Echocardiography (VisualSonics Vevo F2), Force frequency (LabChart8), Ca2+ sparks, fibertyping, and cross-sections (Zeiss LSM 510), Mass Spectrometry (nLC 1200 and Orbitrap Fusion mass spectrometer, Thermo Scientific).
Data analysis	Echocardiography (Vevo Lab 5.7.1), Force frequency (Chart5 version 5.2), Immunoblot (ImageJ 1.5, NIH), Ca2+ sparks (Auto-TT), fibertyping and cross-sections (Myosight), Mass Spectrometry (Proteome Discoverer 2.1 interface PD 2.5, Thermo Fisher) and (Mascot 2.4, Matrix Science).<br>Statistics for null hypothesis testing (Prism 10.0, GraphPad) and power calculation (RStudio 1.0.143, R).

For manuscripts utilizing custom algorithms or software that are central to the research but not yet described in published literature, software must be made available to editors and reviewers. We strongly encourage code deposition in a community repository (e.g. GitHub). See the Nature Portfolio guidelines for submitting code & software for further information.

Data

Policy information about availability of data

All manuscripts must include a data availability statement. This statement should provide the following information, where applicable:

- Accession codes, unique identifiers, or web links for publicly available datasets
- A description of any restrictions on data availability
- For clinical datasets or third party data, please ensure that the statement adheres to our policy

All data generated or analyzed during this study are included in this published article and its supplementary information files. The mass spectrometry data will be deposited via the Mass Spectrometry Interactive Virtual Environment (MassIVE) repository. The plasmids are publicly available through Addgene. Source data are provided with this paper and open to the public.

Research involving human participants, their data, or biological material

Policy information about studies with human participants or human data. See also policy information about sex, gender (identity/presentation), and sexual orientation and race, ethnicity and racism.

Reporting on sex and gender	This study did NOT involve human participants.
Reporting on race, ethnicity, or other socially relevant groupings	This study did NOT involve human participants.
Population characteristics	This study did NOT involve human participants.
Recruitment	This study did NOT involve human participants.
Ethics oversight	This study did NOT involve human participants.

Note that full information on the approval of the study protocol must also be provided in the manuscript.

Field-specific reporting

Please select the one below that is the best fit for your research. If you are not sure, read the appropriate sections before making your selection.

☒ Life sciences ☐ Behavioural & social sciences ☐ Ecological, evolutionary & environmental sciences

For a reference copy of the document with all sections, see nature.com/documents/nr-reporting-summary-flat.pdf

Life sciences study design

All studies must disclose on these points even when the disclosure is negative.

Sample size	Sample sizes were determined based on power analysis of preliminary results or estimated based on similar previous studies when preliminary results were not available.
Data exclusions	No samples were excluded from the statistical analysis unless specifically stated otherwise.
Replication	The number of biological replicates (sample sizes) for each experiment was indicated. All key data were retrieved from at least 3 independent experiments.
Randomization	For experiment involving animal subjects, mice were allocated into experimental groups with a specifically designed randomization mechanism. Unique animal identifiers (mating cage number/litter number/earmark/ID) were assigned to each mouse at weaning based on a fully randomized order of selection from a mating cage. Littermate pairs were required for all experiments in our study to control for covariates influenced by any genetic and environmental factors.
Blinding	We confirm that the investigators were blinded for all data collection and analysis. Blinding was done by an independent investigator who randomized and masked the animal information prior to assessment.

Reporting for specific materials, systems and methods

We require information from authors about some types of materials, experimental systems and methods used in many studies. Here, indicate whether each material, system or method listed is relevant to your study. If you are not sure if a list item applies to your research, read the appropriate section before selecting a response.

Materials & experimental systems

n/a	Involved in the study	
☐	☒	Antibodies
☒	☐	Eukaryotic cell lines
☒	☐	Palaeontology and archaeology
☐	☒	Animals and other organisms
☒	☐	Clinical data
☒	☐	Dual use research of concern
☒	☐	Plants

Methods

n/a	Involved in the study	
☒	☐	ChIP-seq
☒	☐	Flow cytometry
☒	☐	MRI-based neuroimaging

Antibodies

Antibodies used

The information on the list of antibodies used can be found in Supplementary Table 3.

Validation

The following primary antibody validation information can be found in the manufacturer's website:

1. Anti-Cav1.2α1 antibody (ACC-003) from Alomone Labs: Rabbit polyclonal anti-Cav1.2α1 antibody for mouse samples. Validated for immunoblotting applications. Cited in 165 publications.

2. Anti-Cav1.1α1 antibody (MA3-920) from Thermo Scientific: Mouse monoclonal anti-Cav1.1α1 antibody for mouse samples. Validated for immunoblotting applications. Cited in 38 publications.

3. Anti-Jph1 antibody for aa559-572 (40-5100) from Thermo Scientific: Rabbit polyclonal anti-Jph1 antibody for mouse samples. Validated for immunoblotting applications. Cited in 7 publications.

4. Anti-Jph2 antibody for aa1-50 (MA5-32864) from Invitrogen: Mouse monoclonal anti-Jph2 antibody for mouse samples. Validated for immunoblotting applications.

5. Anti-Jph2 antibody for aa565-580 (40-5300) from Invitrogen: Rabbit polyclonal anti-Jph2 antibody for mouse samples. Validated for immunoblotting applications. Cited in 10 publications.

6. Anti-Jph2 antibody for aa431-680 (sc-377086) from Santa Cruz: Mouse monoclonal anti-Jph2 antibody for mouse samples. Validated for immunoblotting applications. Cited in 9 publications.

7. Anti-RyR antibody (34C) from DSHB: Mouse monoclonal anti-RyR antibody for mouse samples. Validated for immunoblotting and immunoprecipitation applications. Cited in 14 publications.

8. Anti-Speg antibody (12472-T16) from Sino Biological: Rabbit polyclonal anti-Speg antibody for mouse samples. Validated for immunoblotting applications. Cited in 3 publications.

9. Anti-MyHC I antibody (BA-F8) from DSHB: Mouse monoclonal anti-MyHC I antibody for mouse samples. Validated for immunofluorescence applications. Cited in 24 publications.

10. Anti-MyHC IIa antibody (SC-71) from DSHB: Mouse monoclonal anti-MyHC IIa antibody for mouse samples. Validated for immunofluorescence applications. Cited in 42 publications.

11. Anti-MyHC IIb antibody (BF-F3) from DSHB: Mouse monoclonal anti-MyHC IIb antibody for mouse samples. Validated for immunofluorescence applications. Cited in 31 publications.

12. Anti-Laminin antibody (ab11575) from Abcam: Rabbit polyclonal anti-laminin antibody for mouse samples. Validated for immunofluorescence applications. Cited in 23 publications.

13. Anti-Fsd2 antibody (25609-1-AP) from Proteintech: Rabbit polyclonal anti-Fsd2 antibody for mouse samples. Validated for immunoblotting applications.

14. Anti-Esd antibody (PA5-96537) from Invitrogen: Rabbit polyclonal anti-Esd antibody for mouse samples. Validated for immunoblotting applications.

15. Anti-HA antibody (66006-2-Ig) from Proteintech: Mouse monoclonal anti-HA antibody for mouse samples. Validated for immunoblotting applications. Cited in 93 publications.

Animals and other research organisms

Policy information about studies involving animals; ARRIVE guidelines recommended for reporting animal research, and Sex and Gender in Research

Laboratory animals

Mouse (mus musculus) maintained on C57BL6/J genetic background were used in this study. Detailed gender and age information of mice were specified for each experiment in the manuscript.

Wild animals	This study did NOT involve wild animals.
Reporting on sex	Sex was considered in study design and sex was assigned based on external genitalia of pups upon weaning.<br>Both male and female mice were used when characterizing the functional consequences of Speg deficiency and were analyzed separately. Given the expense of the experiments, the proteomic data were generated primarily with male mice.
Field-collected samples	This study did NOT involve samples collected from the field.
Ethics oversight	The institutional animal care and use committee (IACUC) at Baylor College of Medicine approved all protocols for animal experiments in this study.

Note that full information on the approval of the study protocol must also be provided in the manuscript.

This checklist template is licensed under a Creative Commons Attribution 4.0 International License, which permits use, sharing, adaptation, distribution and reproduction in any medium or format, as long as you give appropriate credit to the original author(s) and the source, provide a link to the Creative Commons license, and indicate if changes were made. The images or other third party material in this article are included in the article's Creative Commons license, unless indicated otherwise in a credit line to the material. If material is not included in the article's Creative Commons license and your intended use is not permitted by statutory regulation or exceeds the permitted use, you will need to obtain permission directly from the copyright holder. To view a copy of this license, visit http://creativecommons.org/licenses/by/4.0/
